# An Implantable Peripheral Nerve Recording and Stimulation System for Experiments on Freely Moving Animal Subjects

**DOI:** 10.1038/s41598-018-24465-1

**Published:** 2018-04-17

**Authors:** Byunghun Lee, Mukhesh K. Koripalli, Yaoyao Jia, Joshua Acosta, M. S. E. Sendi, Yoonsu Choi, Maysam Ghovanloo

**Affiliations:** 1 Georgia Institute of Technology, School of Electrical and Computer Engineering, Atlanta, 30308 USA; 2 University of Texas, Rio Grande Valley, Department of Electrical Engineering, Edinburg, 78539 USA; 3 Incheon National University, Department of Electrical Engineering, Incheon, 22012 South Korea

## Abstract

A new study with rat sciatic nerve model for peripheral nerve interfacing is presented using a fully-implanted inductively-powered recording and stimulation system in a wirelessly-powered standard homecage that allows animal subjects move freely within the homecage. The Wireless Implantable Neural Recording and Stimulation (WINeRS) system offers 32-channel peripheral nerve recording and 4-channel current-controlled stimulation capabilities in a 3 × 1.5 × 0.5 cm^3^ package. A bi-directional data link is established by on-off keying pulse-position modulation (OOK-PPM) in near field for narrow-band downlink and 433 MHz OOK for wideband uplink. An external wideband receiver is designed by adopting a commercial software defined radio (SDR) for a robust wideband data acquisition on a PC. The WINeRS-8 prototypes in two forms of battery-powered headstage and wirelessly-powered implant are validated *in vivo*, and compared with a commercial system. In the animal study, evoked compound action potentials were recorded to verify the stimulation and recording capabilities of the WINeRS-8 system with 32-ch penetrating and 4-ch cuff electrodes on the sciatic nerve of awake freely-behaving rats. Compared to the conventional battery-powered system, WINeRS can be used in closed-loop recording and stimulation experiments over extended periods without adding the burden of carrying batteries on the animal subject or interrupting the experiment.

## Introduction

Neural interfacing systems in general, and particularly those at the periphery, have significantly benefited from recent advancements in technologies for safe communication pathways with the external world. The acquired data from efferent nerves and motor potential in the peripheral nervous system (PNS) is interpreted to provide meaningful behavior patterns, a variety of sensing feedbacks^1–3^, or to monitor restoration of motor and sensory functions in patients with spinal cord injuries^4–6^. The relatively new concept of “electroceuticals,” which suggests using electrical stimulation of peripheral nerves that control various organs, such as heart and lever, as a substitute for traditional drugs, to achieve a desired outcome, such as adjusting the blood pressure or glucose level, has generated new interest in interfacing with PNS^7–9^. This trend has resulted in high demand for advanced wireless neural interface, which have a more clear pathway towards clinical application, by neuroscientists over their conventional hardwired counterparts, which cause tethering effects, irritation, and potential infection in the host^2,10^. Several battery-powered recording and stimulation devices have been developed and used for experiments on freely-moving animal subjects with limited success. These devices provide a better environment for subjects than the hardwired devices. However, they suffer from the compromise between size of battery and duration of the experiment or functionality of the device, particularly in high channel-count recording and stimulation setups^11–17^. In addition, the battery replacement or recharging becomes an even larger challenge in implantable devices.

To meet the strong demand for research on the nervous system in freely behaving animals within enriched experimental arena without time constraints, several prototypes of wirelessly-powered neural interfaces^12–23^ and wirelessly-powered experimental arena have been developed^23–30^ either for recharging batteries or directly powering neural interfacing devices through inductive links^12–23^, some of which have also been verified *in vivo* with headstage^23,24,31,32^ or implants with somewhat limited functionality^33–35^. In our prior work^24^, a wirelessly-powered homecage system, called EnerCage-HC2, was successfully used to power a wireless and battery-less headstage, which included a one-channel stimulator made of commercial off-the-shelf (COTS) components, for deep brain stimulation (DBS) in rat model. Wirelessly-powered neural interfaces need a receiver (Rx) coil in the mobile unit with sufficient area to harvest enough power to support continuous recording and stimulation operation. This requirement is less challenging to address in headstages^23,24,31,32^ being outside of the animal body. However, the peripheral nerve interfaces, implanted in the animal body, should be much smaller in size, resulting in limitation in the power budget, which in turn affect the implant functionality, such as a small number of channels for recording or very simple stimulation capability, often without a bidirectional data communication^33–35^. Although backpacks can be utilized for providing transcutaneous power, the animal subject will need to carry external bulky devices, including heavy batteries, during the experiment^32,36^.

Here we present an inductively-powered implant with high functionality for a 32-ch peripheral nerve recording and 4-ch current-controlled neural stimulation (CCS), which is also fully compatible with the EnerCage-HC2 system. We have also demonstrated the performance of this system *in vivo* in a study that involved interfacing with the sciatic nerve in the rat model. The proposed presented Wireless Implantable Neural Recording and Stimulation (WINeRS) system is also equipped with bidirectional data communication through a 433 MHz on-off-keying (OOK) transmitter (Tx) for the wideband uplink that carries the recorded neural data, and pulse position modulation (OOK-PPM) based clock and data recovery (CDR) for the narrow band downlink that provides data for stimulation parameters.

**Figure 1:**
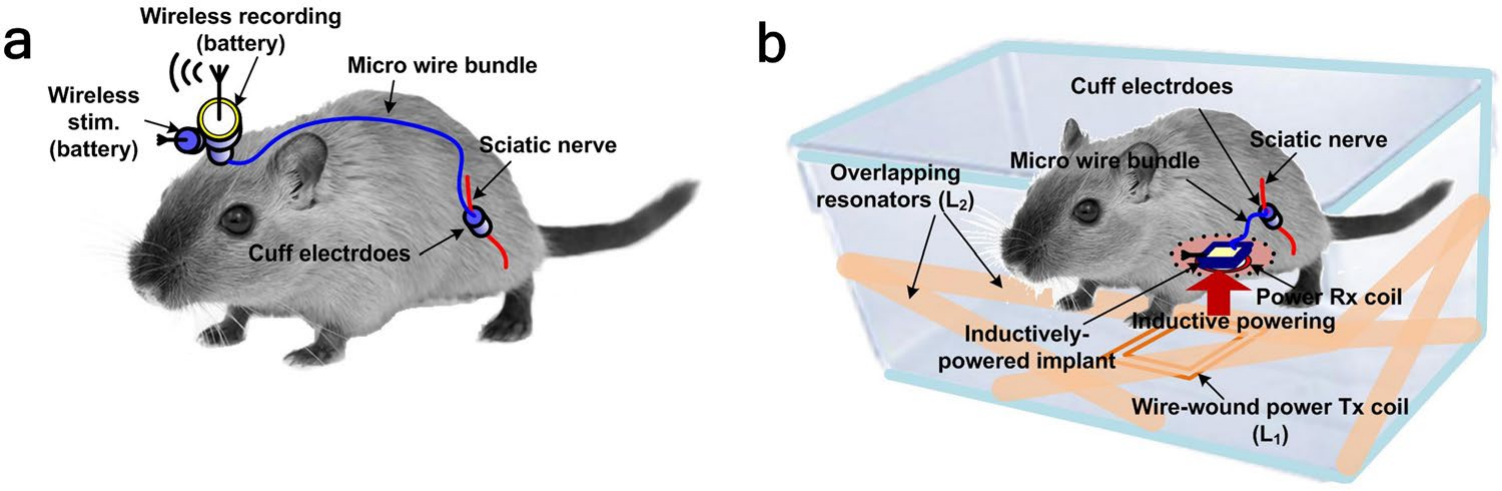
Comparison between two configurations of an animal study involving peripheral nerve interface with sciatic nerve in rat model: (**a**) conventional battery-powered recording/stimulation headstages^6^, and (**b**) inductively-powered WINeRS-8 system in the wirelessly-powered EnerCage-HC2 arena.

The conventional peripheral nerve interface in this animal study using hardwired and battery-powered devices, shown in Fig. 1a, are replaced with the inductively-powered WINeRS-8 system in addition to an automated animal tracking feature inside the standard homecage, as shown in Fig. 1b. The new system offers several advantages in the study of the sciatic nerve in terms of enabling long-term *in vivo* experiments without requiring the animal to carry batteries, lowering the risk of infections by eliminating the headstage transcutaneous connector, and eliminating the potential damage to the surrounding tissue by the long microwire bundle that was needed to connect the target peripheral nerves to the headstage, in addition to reducing the time and complexity of the surgical procedure^6^. The proposed system creates an enriched environment inside the standard homecage for inductively-powered peripheral nerve experiments without adding the burden of carrying large payloads by the freely behaving animal subjects, while eliminating long cables both inside and outside the animal body.

## Results

### System architecture of the WINeRS-8 system to be used in the wirelessly-powered EnerCage-HC2 system

**Figure 2:**
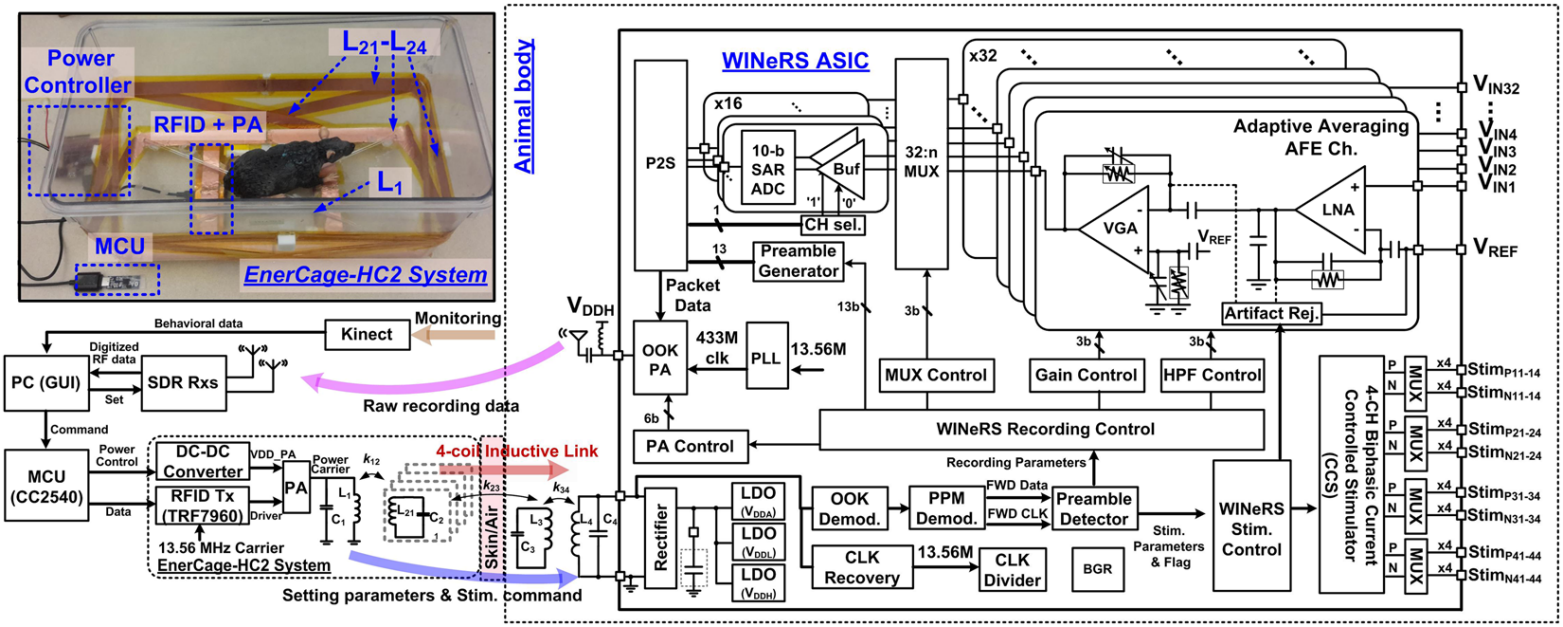
A simplified schematic diagram of the entire system that allows wireless power and data transmission between WINeRS-8 system with 32-ch recording and 4-ch stimulation implanted in a small animal subject, freely behaving in the EnerCage-HC2 smart wirelessly-powered homecage. Inset: a fully-functional prototype with a toy rat.

A simplified schematic diagram of the entire system, including EnerCage-HC2 as the stationary unit and WINeRS-8 system as the mobile unit implanted in the subject body is presented in Fig. 2 (and Fig. S1). This figure also indicates the wireless power and data transmission flows. In EnerCage-HC2, a power amplifier (PA) drives the Tx coil, *L*_1_, to generate an electromagnetic carrier signal at 13.56 MHz, which is a band approved by the Federal Communications Commission (FCC) for industrial, scientific, and medical (ISM) applications. A microcontroller (CC2540) adjusts the PA output power through a DC-DC converter that controls the PA supply voltage (VDD_PA) based on the amount of received power in the mobile unit. This closed-loop mechanism regulates the implant received power at a desired level. The PA can also modulate the power carrier to send data from a PC to the implant (downlink) through the same 4-coil inductive link that powers the implant, for which the OOK-PPM is adopted for its simplicity and reliability to set the CCS parameters. In addition to the driving coil, *L*_1_, the EnerCage-HC2 system has four overlapping resonators, *L*_21_-*L*_24_, wrapped around the standard homecage^24^. During the experiment, a Microsoft Kinect camera captures the animal movements inside the experimental arena with a Red-Green-Blue (RGB, 2D) color camera and an infrared (IR, 3D) depth camera, and delivers the raw data to the PC for automated animal tracking and behavior recognition using several features that are extracted from the 2D-color and 3D-depth images in real time^37^.

To make the implant as compact as possible, WINeRS-8 system is implemented on a chip (SoC) in the form of an application-specific integrated circuit (ASIC) with a handful of off-chip components, as shown in Fig. 2. The 13.56 MHz power carrier is harvested by *L*_4_*C*_4_-tank, rectified, and regulated by three low drop-out (LDO) regulators, which provide low analog (*V*_*DDA*_ = 1 V), low digital (*V*_*DDL*_ = 1 V), and high analog/digital (*V*_*DDH*_ = 2 V) supplies for the rest of the SoC. Clock recovery extracts a reference clock from the 13.56 MHz power carrier and OOK-PPM demodulator recovers the incoming data bit stream that is modulated on the power carrier to set the WINeRS-8 stimulation and other adjustable parameters. An adaptive averaging method in the 32-ch analog front-end (AFE) uses a 32-to-*n* analog multiplexer (MUX) to further reduce the noise by averaging multiple channels for the peripheral nerve recording application, and each low noise amplifier (LNA) has a dc-coupled structure that maintains high input impedance in the order of 61 MΩ at 1 kHz^38^. Every two LNAs share a 50 kS/s 10-bit SAR ADC for digitization, resulting in 25 kS/s per channel. A phase-locked loop (PLL) generates a 433 MHz data carrier from the 13.56 MHz reference clock. Digitized raw data packets are combined with a 13-bit preamble through a parallel-to-series (P2S) conversion block, and control a PA that modulates the 433 MHz data carrier at a rate of 9 Mbps with 176-bit data packets to generate the uplink OOK signal, which in turn drives a small Tx antenna through a matching circuit. This 433 MHz OOK data carrier is picked up outside the homecage by a pair of software defined radio (SDR) receivers (Rx) that are designed to create a robust RF data communication link against freely behaving animal movements inside the homecage during the experiment.

### Closed-loop recording and stimulation capability

**Figure 3:**
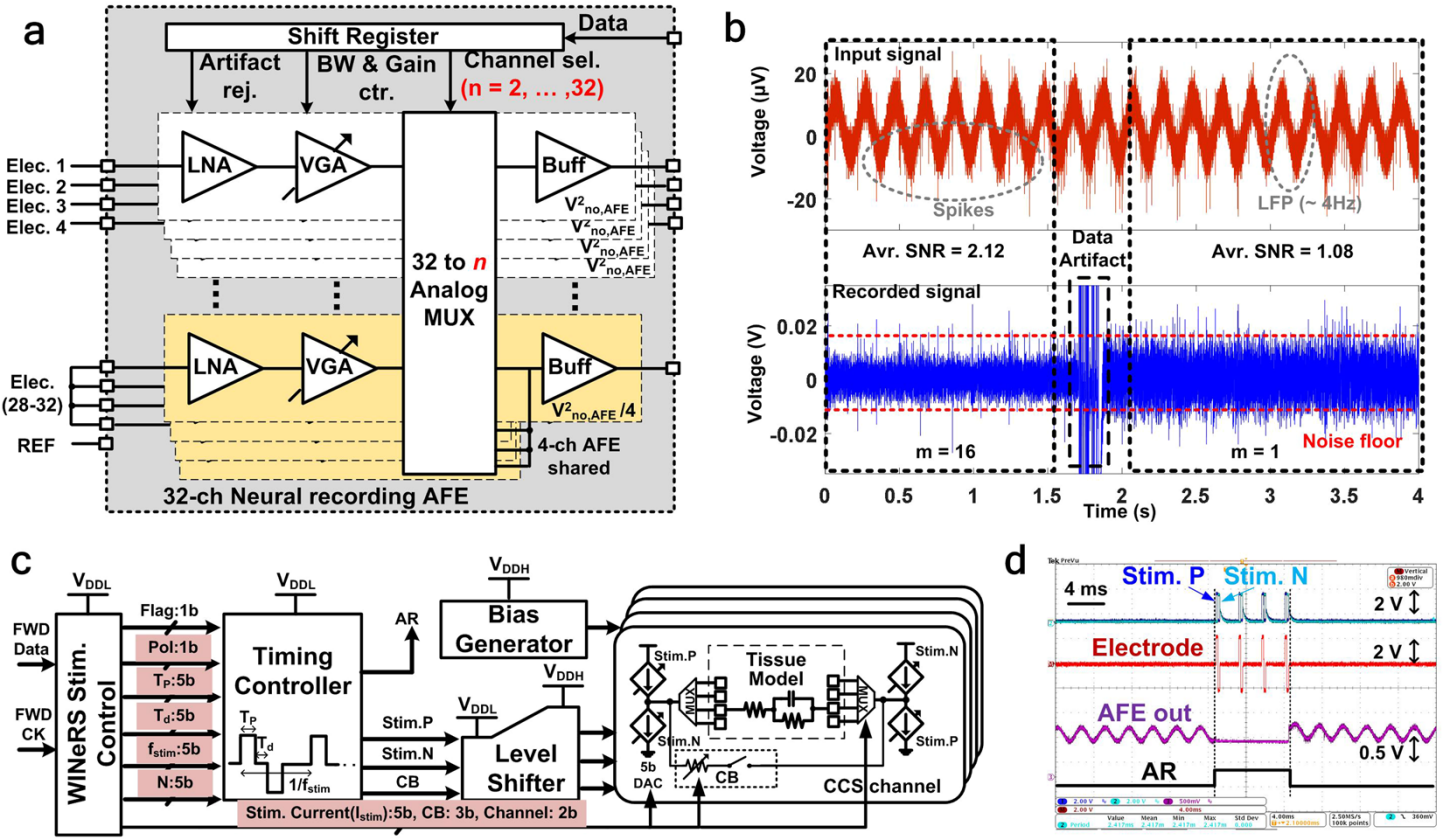
(**a**) Simplified schematic diagram of 32-ch AFE with adaptive averaging technique for peripheral nerve recording, (**b**) a pre-recorded spike signal (Top) and resulting AFE output signal (Bottom) with transient noise variation from *m* = 16 to *m* = 1 for SNR comparison, (**c**) schematic diagram of 4-ch biphasic CCS for simultaneous peripheral nerve recording and stimulation, and (**d**) *in-situ* experiment for biphasic stimulation and stimulus artifact rejection using Randles equivalent tissue model^39^.

The adaptive averaging method^38^, which schematic is shown in Fig. 3a, reduces the input referred noise by combining *m*-channels as,

1 $${v}_{ni\_rms,AFE,m}=\frac{{v}_{ni\_rms,AFE,1}}{\sqrt{m}}$$

where *m* is *32/n*, *n* = *2*, 4*,… 32*, and *V*_*ni_rms,AFE,1*_ is the input referred noise of each AFE channel. Figure 3b compares a pre-recorded spike signal, which includes attenuated ~30 μV_pp_ spikes added to 4 Hz sinusoid interference, representing large electromyography (EMG) signal. The adaptive averaging method can increase the signal-to-noise ratio (SNR) and filter out the emulated EMG signal. This mechanism offers flexible and higher SNR in peripheral nerve recordings, in which signal strength is typically defined by the electrode-tissue interface.

Figure 3c shows the block diagram of the biphasic CCS with its adjustable stimulation parameters. To increase the voltage compliance within the 2 V supply (*V*_*DDH*_), a biphasic topology is adopted. A timing controller utilizes *V*_*DDL*_ to save power. In total, 30 bits are utilized to define stimulation parameters including: Pol (1 bit) = polarity (positive/negative), *f*_*stim*_ (5-bit) = stimulation frequency (13–414 Hz), *T*_*p*_ (5-bit) = stimulus width (9.5–304 μs), *T*_*d*_ (5-bit) = stimulus delay (9.5–304 μs), *I*_*Stim*_ (5-bit) = stimulus amplitude (60–1860 μA), *Channel* (2-bit) = Active stimulation channel (1 to 4), *N* (4-bit) = number of stimulus pulses (1–16), and *CB* (3-bit) = charge balancing. The charge balancing (*CB*) pulse can be used to remove the residual charge in the tissue, if the positive and negative stimulation pulses are not perfectly balanced. These parameters are provided by the *WINeRS Stimulator Control* block, which follows the OOK-PPM demodulator, as shown in Fig. 2. The 1-bit stimulation flag signal, *Flag*, is generated by the control block to synchronize the stimulation pulses with the stimulus artifact rejection signal, *AR*. The *AR* pulse is utilized in the AFE to prevent saturation of the LNAs from large stimulus current, and enable the recording function to resume right after the last stimulation pulse, as shown in Fig. 3d. These waveforms show *in-situ* stimulation and stimulus artifact rejection using a Randles equivalent model^39^. Since the generated *AR* signal keeps the AFE channel at the reference voltage during the stimulation, the AFE output shows the ability to resume recording within 0.2 ms after stimulation without being saturation, as shown using the sinusoid signal in Fig. 3d.

### Low power downlink data communication

One of the important features of WINeRS-8 is the downlink data telemetry to control the implant adjustable parameters in real time. Downlink data initiates from the graphical user interface (GUI), running on the PC, and allows the user to set parameters, such as gain and bandwidth of the AFE, RF transmitted power, and aforementioned 30-bit stimulation parameters. Since downlink data telemetry does not need high data rate, near-field communication within the EnerCage-HC2 system was found to be the most suitable method.

**Figure 4:**
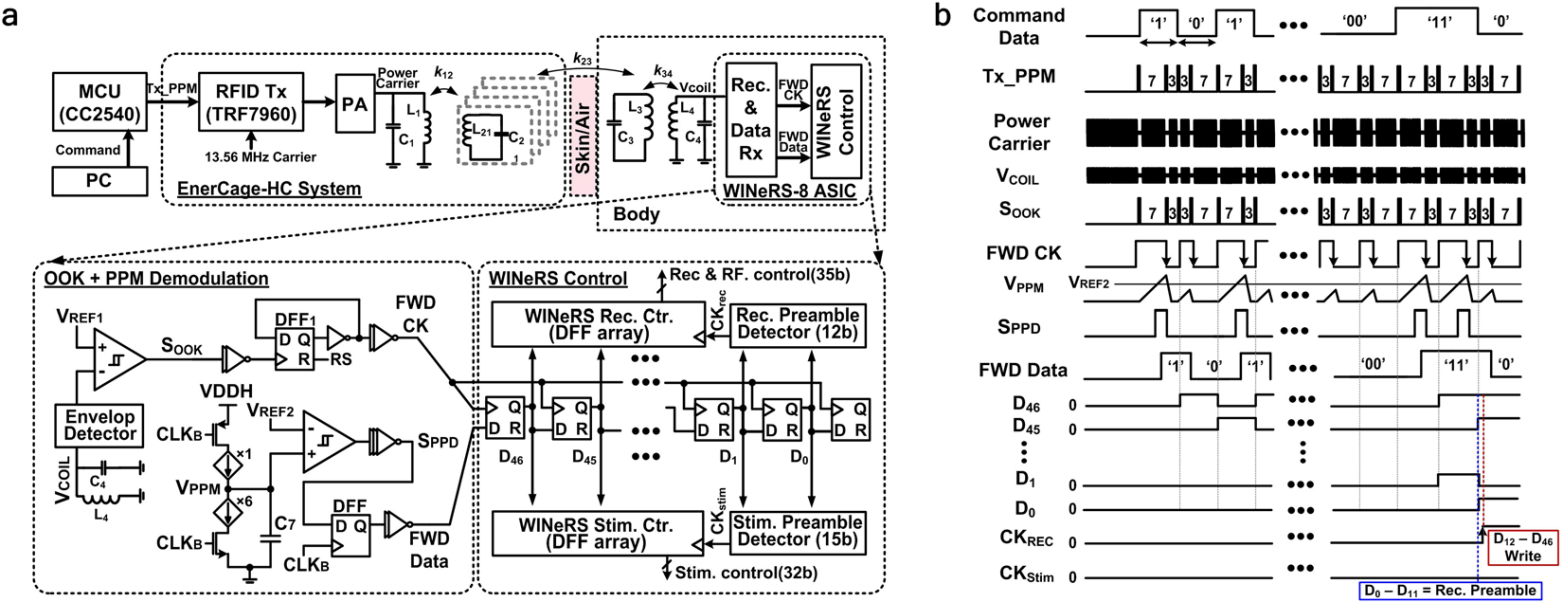
(**a**) Block diagram of the OOK-PPM downlink data transmission between EnerCage-HC2 (Tx) and WINeRS-8 (Rx) systems via 4-coil inductive link, and (**b**) conceptual waveforms of the downlink data at various nodes of the block diagram.

Although amplitude shift keying (ASK) is the most popular method because of its simple modulation and demodulation circuitry and low power consumption^40^, synchronization between data and clock signals render ASK sensitive to the inductive coupling, k_23_ in Fig. 2, variation because of the animal movements in the homecage and existing noise in the power carrier amplitude. Therefore, we combined PPM with OOK to be able to recover the clock and synchronized data, which are necessary for reliable communication^41^. In Fig. 4a, the control commands in the PC are converted to the corresponding PPM pulses (*Tx_PPM*) via MCU (CC2540). An RFID reader (TRF7960) generates the PPM-OOK-modulated power carrier signal, *f*_*p*_ = 13.56 MHz, which is used for both wireless powering and downlink data transmission. The received power carrier at the Rx *L*_4_*C*_*4*_-tank, *V*_*coil*_, is filtered by an envelope detector, and OOK pulses are recovered by threshold detection, when compared against *V*_*REF1*_. The recovered OOK pulses, *S*_*OOK*_, repeatedly charge and discharge the integration capacitor, *C*_7_, depending on the delay between the individual OOK pulses, while the synchronized data clock, *FWD CK*, is extracted from the edges of the OOK pulses. When *V*_*PPM*_ at *C*_7_ goes above *V*_*REF2*_, the comparator output, *S*_*PPD*_, is set to ‘1’ and the recovered data, *FWD Data*, is recognized as ‘1’ while being in sync with *FWD CK*, as shown in Fig. 4b.

*FWD Data* is shifted in the data buffer, which consists of 47 D-type flip-flops (DFFs), to detect either the Rec/RF preamble or stimulation preambles in two separate preamble detectors. The stimulation preamble has 15 bits, while the Rec/RF preamble has 12 bits. As shown in Fig. 4b (and Fig. S2), if the shifted *FWD Data*, *D*_0_*-D*_11_, is matched with predefined 12-bit Rec/RF preamble, *CK*_*REC*_ flag is triggered and the following data, *D*_12_*-D*_46_, is latched for control of the recording and RF Tx blocks. Similarly, if *D*_0_*–D*_14_ are matched with the predefined 15-bit stimulation preamble, *CK*_*Stim*_ flag is triggered to activate the stimulation block with the given stimulation parameters in the following data bits that were shown in Fig. 3c.

### Wideband uplink data transmission for recording raw neural signals

**Figure 5:**
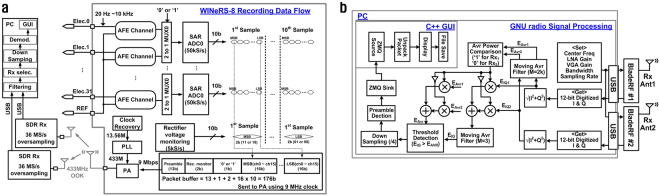
(**a**) Block diagram of the uplink data Tx from the 32-ch MEA to the PC via 433 MHz OOK Tx in the WINeRS-8 SoC, and (**b**) flowchart of the external dual SDR Rx for extended coverage of the experimental arena and elimination of blind spots.

The focus of most high performance wireless neural interfaces in literature is on the wideband RF data transmission, which typically relate to the implantable Tx side of the system. However, a complete wireless data acquisition system also needs wideband Rx antenna(s), external RF front end, high speed PC interface, post processing, storage, and data visualization on a GUI, particularly if the system is supposed to operate in real time^14^. Figure 5a shows the block diagram of the data transmission block from 32-ch microelectrode array (MEA) to the PC, through the 433 MHz OOK Tx in WINeRS-8 SoC. The neural signal from every channel has a bandwidth of 10 kHz, and SAR ADC digitizes the LNA output at 25 kS/s. The rectifier voltage is also sampled at 5 kS/s to monitor the status of the inductive powering and close the power transmission loop. A 176-bit packet data is delivered to the OOK PA at a rate of 9.04 MHz to modulate the 433 MHz RF data carrier, generated by the PLL. The RF signal from the WINeRS-8 Tx is picked up by two Rx antennas placed outside the homecage to extend the wireless coverage of the experimental arena and eliminate any blind spots caused by the Tx antenna directivity. The received signals are amplified/filtered independently through a pair of parallel SDR RF front-ends, which also digitize and send them to the PC via separate USB ports, where a custom algorithm implemented in GNU radio^42^ and C++ GUI perform post processing and demodulation of the incoming RF data stream from the SDRs, respectively, to display the recovered neural signals on the PC screen in real time, and store it on the hard disk.

Flowchart of the dual-SDR WINeRS-8 Rx is shown in Fig. 5b. Two BladeRF SDRs (Nuand LLC, Rochester, NY) are utilized in the WINeRS-8 Rx, which cover 300 MHz–3.8 GHz RF spectrum and are equipped with full duplex, 12 bit ADC with 40 MS/s sampling rate and field-programmable gate array (FPGA) logic^43^. A moving average filter (M = 2k) compares the average power of the received signal from the individual Rx antennas, and decides which SDR Rx has a better signal to noise ratio (SNR). Data from the SDR Rx data with higher SNR is demodulated and sent to the GUI software. This method can be easily extended to multiple SDR Rx to achieve robust wideband RF data recovery over larger experimental arenas without any blind spots.

### Prototypes of the WINeRS-8 system

**Figure 6:**
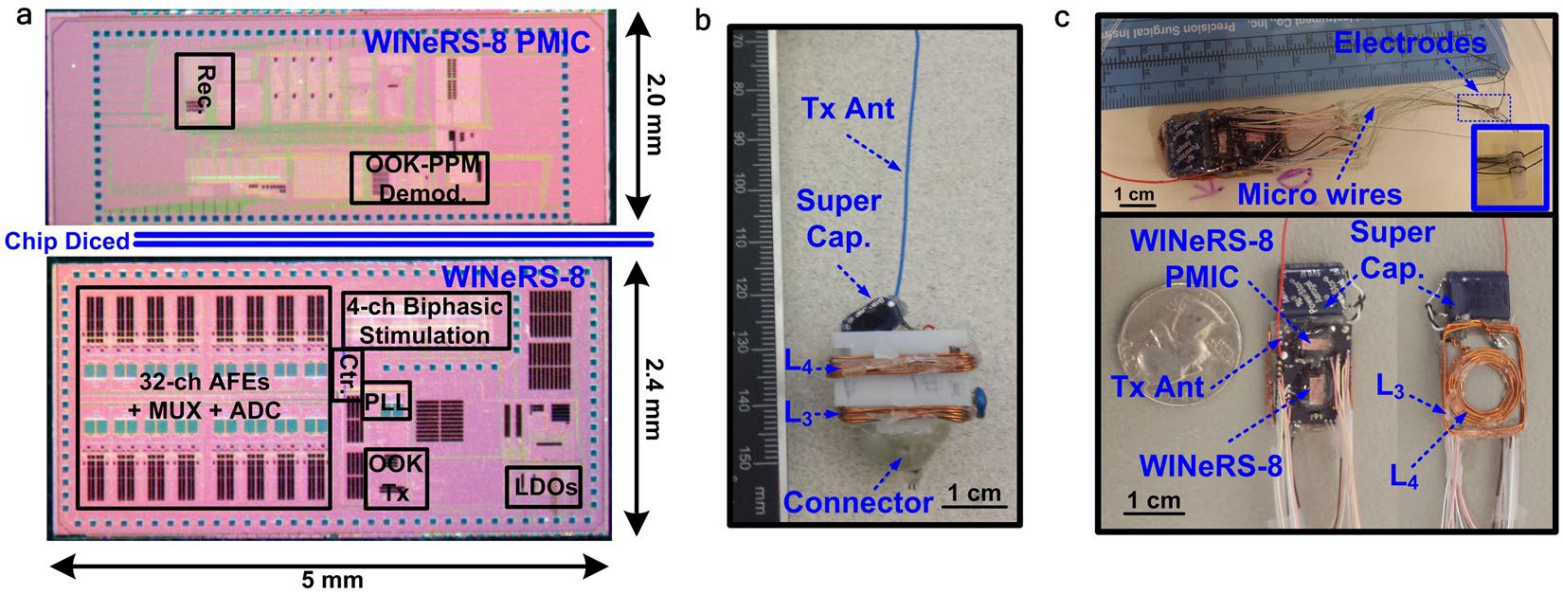
(**a**) Die photo, (**b**) headstage, and (**c**) implant prototypes of the inductively-powered WINeRS-8 SoC for *in vivo* experiments involving freely behaving animals within the EnerCage-HC2 system.

WINeRS-8 SoC has been designed and implemented in 130-nm standard CMOS technology and occupies 5 × 4.4 mm^2^, as shown in Fig. 6a. In order to validate the functionality of the WINeRS-8 system *in vivo* and compare its usage forms, two prototypes, one a conventional headstage (Fig. 1a) and the other an implant (Fig. 1b), were constructed, as shown in Fig. 6b,c, respectively, and used to interface with the sciatic nerve in rat animal model. WINeRS-8 prototypes were both equipped with 0.21 F super-capacitors as buffers that supply the SoC when the received power was temporarily interrupted by certain movements of the animal subject. Each device has *L*_3_ and *L*_4_ coils optimized for the highest power transfer efficiency (PTE) inside the EnerCage-HC2^24^. A third prototype was also constructed, in which the super-capacitor was replaced with a 100 mAh battery to allow conducting experiment outside the EnerCage-HC2. Instead of the OOK-PPM downlink for setting parameters, a COTS MCU (CC2541) with built-in Bluetooth low energy (BLE) wireless link was utilized in this version (Fig. S3). A monopole antenna was utilized for RF data Tx, which can be wrapped around the implant. The power management integrated circuit (PMIC) block of the WINeRS-8 SoC in this prototype was diced for reducing the prototype width. The resulting WINeRS-8 implant dimensions were 3 × 1.5 × 0.5 cm^3^ and weighed 2.8 g, including the super-cap. The nominal power consumption of the device was 18.9 mW.

### *In vivo* test setup

**Figure 7:**
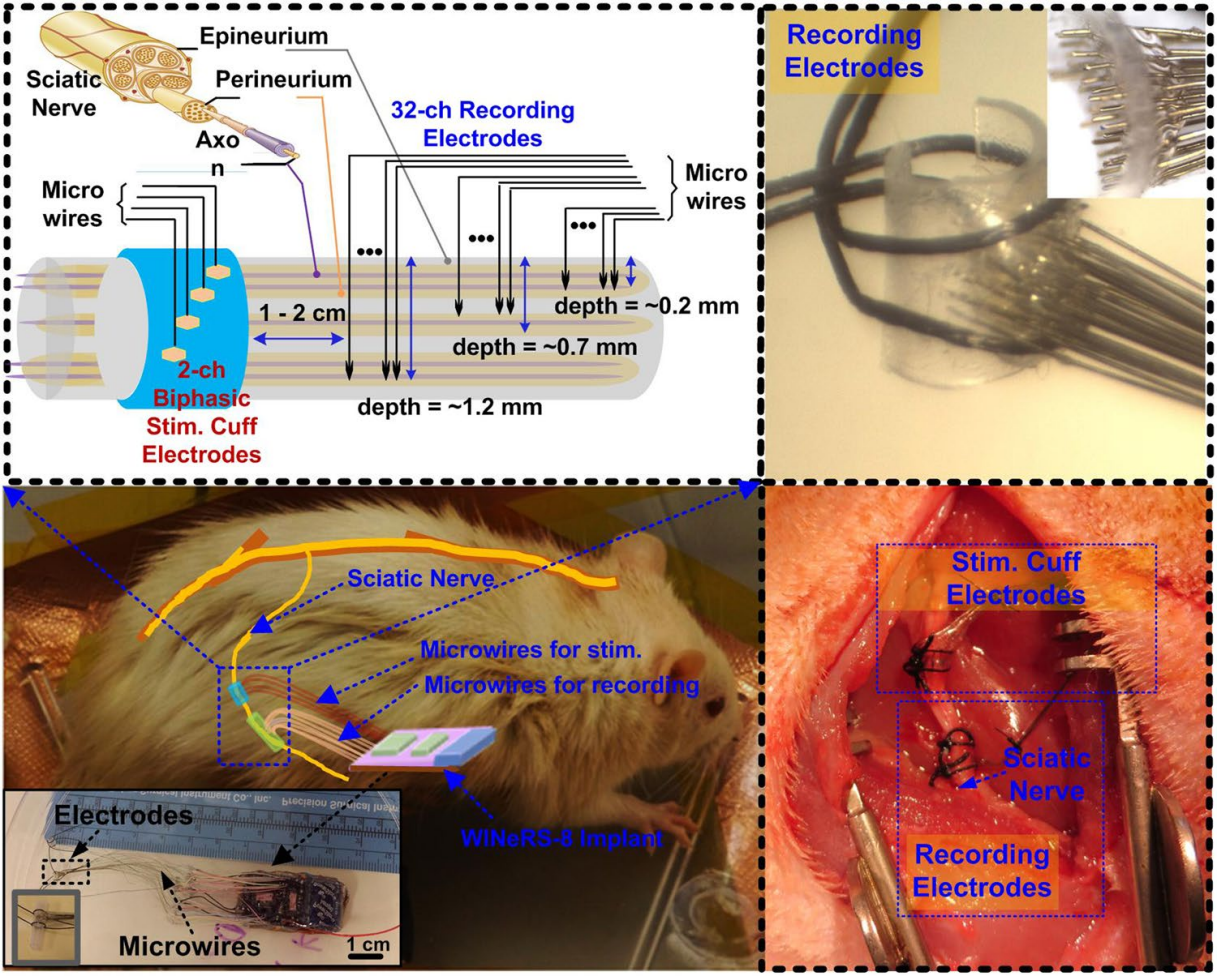
The rat sciatic nerve representation with stimulation and recording electrodes. 32-ch penetrating MEA and 2-ch bipolar cuff electrodes are connected to the WINeRS-8 implant via microwires.

After verifying the headstage version of WINeRS-8, the implant version was implanted in separate animals, and evoked compound action potentials were recorded to verify the WINeRS-8 system recording and stimulation capabilities. Two different types of MEAs were utilized, cuff MEA for stimulation and penetrating microwire MEA for recording, as presented in Fig. 7. The cuff electrodes for 2-ch bipolar stimulation are placed 1–2 cm from the penetrating recording MEA and distal with respect to the spinal cord. The penetrating MEA is composed of three layers, at 0.2 mm, 0.7 mm, and 1.2 mm depth, considering the typical thickness of Epineurium and Perineurium in the nerve. Since these recording electrodes still are in contact with the penetrated axons, their recordings become highly correlated. The penetrating MEA and biphasic cuff electrodes on the sciatic nerve were connected to a 75 µm thick microwire bundles (Stablohm 800 A, California Fine Wire).

The following *in vivo* experiments were conducted for verification of the WINeRS-8 SoC functionality for peripheral nerve interfacing, as shown in Fig. 1. Five female Lewis rats are used for the *in vivo* experiments when they had reached 12 weeks of age and at least 220 g of weight. All experiments are conducted with prior approvals from the Institutional Animal Care and Use Committee (IACUC) at University of Texas Rio Grande Valley and Georgia Institute of Technology.

1. Recording and stimulation using a combination of two battery-powered commercial headstages, a w-32 wireless recording headstage and a S2W stimulator headstage (TBSI, Durham, NC), as shown in Fig. 8a.

**Figure 8:**
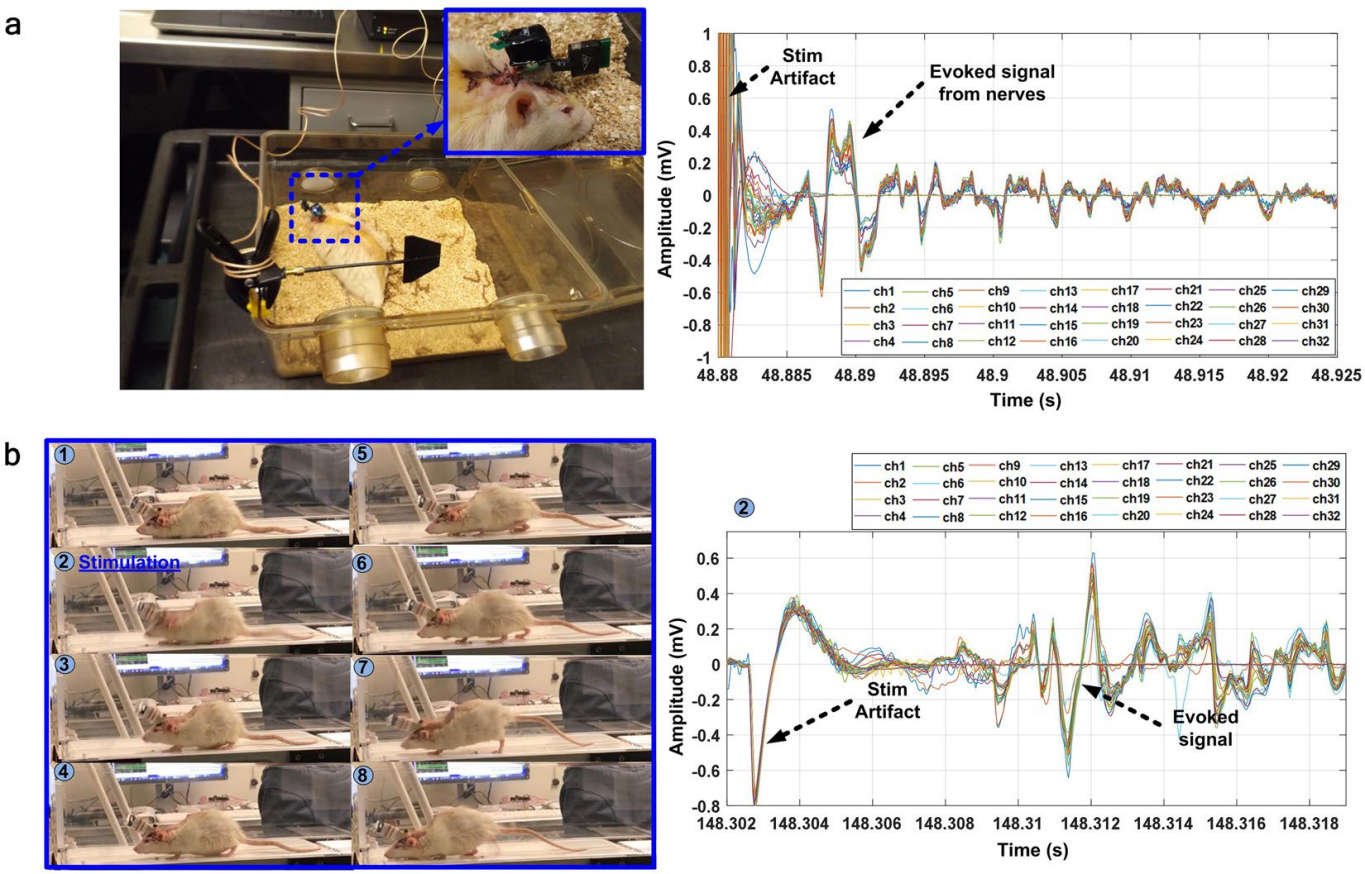
Battery-powered *in vivo* experiment setup. (**a**) Commercial recording/stimulation headstages, and (**b**) WINeRS-8 headstage used outside the homecage for measuring evoked compound signals from the rat sciatic nerve during treadmill locomotion.
2. Recording and stimulation on a treadmill using the battery-powered WINeRS-8 headstage, as shown in Fig. 8b.
3. Recording and stimulation inside the EnerCage-HC2 system using the inductively-powered WINeRS-8 headstage, as shown in Fig. 9b.

**Figure 9:**
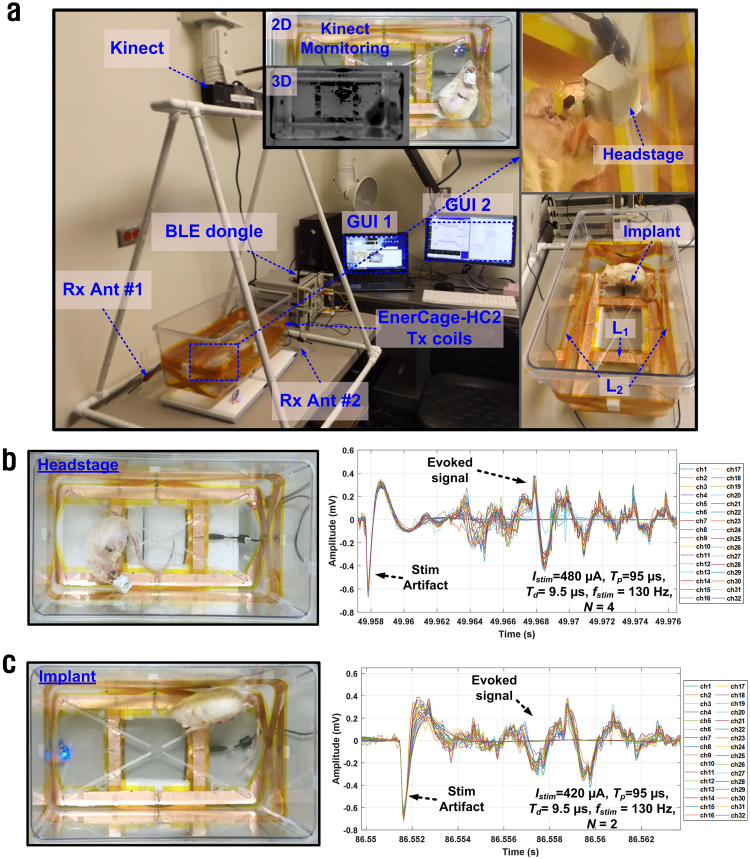
(**a**) Inductively-powered *in vivo* experiment setup for WINeRS-8 headstage and implant versions. Insets show captured images by the Kinect camera. Recorded evoked compound action potential from the sciatic nerve after stimulation, which are shown on the right side using: (**b**) WINeRS-8 headstage, and (**c**) WINeR-8 implant.
4. Recording and stimulation inside the EnerCage-HC2 system using the inductively-powered WINeRS-8 implant, as shown in Fig. 9c.

The *in vivo* experimental setups of the battery-powered commercial and WINeRS-8 headstages are shown in Fig. 8a,b, respectively, together with their measured evoked compound signals from the rat sciatic nerve. During the experiment, the stimulation parameters were changed to observe the effects on rat behavior on the treadmill while observing the recorded evoked signal. In this experiment, the stimulation pulses are periodically delivered to the cuff electrodes to record evoked signals from the recording electrodes, while the animal is walking on the treadmill. The measured waveforms were synchronized with video recordings using an external camera, as a substitute for the Kinect camera function within the EnerCage-HC2 system. When the rat showed distinguishable behavioral response from each stimulation, the evoked signal can be observed in 5–10 ms after the stimulus artifact in both commercial system and WINeRS-8 headstage, considering the conduction velocity of the sciatic nerve when it is injured after the implantation surgery (5~20 m/s)^44,45^. Thanks to the stimulus artifact rejection capability of WINeRS-8 system, described above, the recorded stimulus artifact is around 3.3 times smaller than the commercial headstage combination.

The experimental setup for the inductively-powered WINeRS-8 headstage and implant inside the EnerCage-HC2 system is shown in Fig. 9a. The WINeRS-8 implant in Fig. 6c was implanted in the abdominal cavity of the rat, and the stimulation and recording electrodes were directly connected to the implant using a microwire bundle, insulated by polyimide at the junction. WINeRS-8 headstage, on the other hand, was mounted on the rat head using a 32-pin Omnetics connector (Nano Strip Connector, A79022-001, Omnetics, MN), which was secured on the rat skull using stainless steel mounting screws (00-96 × 1/16, Plastic One, VA) and dental cement. A long microwire bundle connected the Omnetics connector on the head to the sciatic nerve electrodes under the skin across the back of the rat. During the experiment, the Kinect camera, mounted on top of the EnerCage-HC2 (see Fig. 9a) provides automated animal tracking information using images from the 2D-color and 3D- depth cameras^37^. In the headstage experiment, shown in Fig. 9b, the stimulation parameters of *I*_*Stim*_ = 480 μA, *T*_*P*_ = 95 μs, *T*_*d*_ = 9.5 μs, *f*_*Stim*_ = 130 Hz, and *N* = 4 was applied to measure the evoked compound signals after the stimulation, while observing changes in the animal behavior. In the implant experiment, similar response was observed from the rat with stimulation parameters of *I*_*Stim*_ = 420 μA, *T*_*P*_ = 95 μs, *T*_*d*_ = 9.5 μs, *f*_*Stim*_ = 130 Hz, and *N* = 2 with similar patterns of evoked compound signals after the stimulation. During the experiment, the stimulation parameters were changed to observe the animal response and changes in evoked signals from the nerve (Supplementary movie 1). These *in vivo* results demonstrated that WINeRS-8 implant can support functional simultaneous recording and stimulation experiments, while being fully compatible with the EnerCage-HC2 system. The Kinect camera also monitors the animal movements and behavior^37^, evoked by stimulation in real time (Supplementary movie 3).

## Discussion

Although hardwired recording and stimulation devices can provide a large number of channels with good noise performance due to lower size, power, and data constrains, the cable attached to the animal is a limiting factor in natural behavior and cause complications in surgery^10,46–48^. To overcome these limitations, various battery-powered wireless recording and stimulation systems have been developed^11–17^. Although these devices provide wireless recording and stimulation capabilities, battery lifetime remains to be a constraint in long-term experiments. Moreover, battery replacement dramatically increases the possibility of infection in the animal subject with implantable devices. To address this issue, several wirelessly-powered devices have been demonstrated that can charge the implanted battery or directly power the implanted device. However, these devices are separately developed without enough emphasis on the stationary platform, which provides the wireless power, bi-directional data communication, data acquisition backend, visualization, post processing, storage, and behavioral tracking in the experimental arena for conducting long-term electrophysiology experiments. The wirelessly-powered devices in prior works have presented somewhat limited functionality in a small number of applications due to limited number of channels^24,31,32,35^, limited control of stimulation parameters^33,34^, or large device size for implantation and surgery^23,24,31,32^.

Leveraging our prior work on a smart wirelessly-powered homecage, resulting in the EnerCage system^24,30^, we have developed the 8^th^ generation of the Wireless Implantable Neural Recording and Stimulation (WINeRS) system in a way that it is fully compatible with the EnerCage-HC2 for a wide variety of experiments on small freely behaving animals over extended periods. WINeRS-8 SoC is implemented in 130-nm standard CMOS process with minimum off-chip components to reduce the size, while supporting enough recording and stimulation channels at a high noise performance level, needed for peripheral nerve interfacing. In this system, the 32-ch AFE is equipped with an adaptive averaging topology to provide users with a trade-off between the number of channels and input referred noise. The DC-coupled LNA with transconductance input stage has eliminated coupling capacitors without chopper modulation to maintain high input impedance and high SNR, particularly for multichannel PNS recording^38^.

We have adopted two different types of data transmission techniques for simultaneous recording and stimulation, which are the low power narrow band OOK-PPM downlink and wideband OOK data transmission for uplink. Since the uplink data requires continuous high data rate of up to 9 Mbps to transmit the raw digitized neural signals from 32 channels, a complete end-to-end wideband RF data acquisition/transmission paradigm is implemented all the way from MEAs to the PC through dual-SDR Rx with extended coverage area. The output power of the 433 MHz Tx in WINeRS-8 system can be digitally adjusted from the PC in 32 levels, up to 0.2 dBm at 9 Mbps data rate. Thanks to the dual-SDR Rx, which is commercially available, WINeRS-8 Rx is easy to implement, scale up, and modify, compared to our earlier custom-designed wideband WINeR Rx^14^. The GNU radio and customized GUI facilitate RF signal processing, demodulation, and demultiplexing of the incoming data stream, to be ready for real time observation and post-processing on the PC (Supplementary Fig. S4). Downlink data telemetry is achieved by OOK-PPM near field data communication through the EnerCage-HC2 4-coil inductive link to minimize the power consumption and maintain robustness despite coupling variations due to animal movements.

We demonstrated functionality of the WINeRS-8 system *in vivo* for recording from and stimulation of sciatic nerve in freely behaving rats in acute experiments, and compared the evoked compound peripheral nerve signals recorded by battery-powered commercial and inductively-powered WINeRS-8, both in the form of headstage and implanted devices. Implantation of the inductively-powered WINeRS-8 SoC with 32-ch recording and 4-ch stimulation became feasible much closer to the target sciatic nerve (Fig. 6c) thanks to its full integration. Similar evoked compound signal patterns were observed from the sciatic nerve in the commercial battery-powered device, WINeRS-8 headstage, and WINeRS-8 implant prototypes, validating the results. Electrically evoked animal movements following stimulation were also observed (Supplementary movies 1 and 2), and captured by the Kinect tracking system. Unlike battery-powered systems, a combination of WINeRS-8 and EnerCage-HC2 allow for long-term recording and stimulation, enabling fully-automated closed-loop recording and stimulation in future studies. Table 1 compares key specifications of the recording and stimulation devices used in the *in vivo* experiments. To the best of our knowledge, the presented system is the first wirelessly-powered and battery-less device that can be implanted in small freely-behaving animals and used in experiments that involve PNS interfacing with 32-ch recording and 4-ch stimulation capabilities.

**Table 1:** Comparison between the recording/stimulation devices used in the *in vivo* experiments.

Parameters	Commercial devices		WINeRS-8 system		
Type	Headstage (w-32)	Headstage (S2W)	Headstage (WINeRS-8 + MCU)	Headstage (WINeRS-8)	Implant (WINeRS-8)
Power Source	Battery	Battery	Battery	Inductive	Inductive
Function	Neural Recoding	Electrical Stimulation	Recording + Electrical Stimulation		
# of Channel	32	2	32/2		
Gain	800	—	380–6300		
Bandwidth (Hz)	0.8–7 k	—	20–15k		
Input Impedance	6.5 MΩ	—	61 MΩ		
Input referred noise	5.5 µV_rms_	—	3.0 µV_rms_		
Data Carrier	3.05 GHz	—	433 MHz (OOK)/2.3 GHz (BLE)	433 MHz (OOK)/13.56 MHz (OOK-PPM)	
Stimulus Current	—	Up to 1 mA	60 µA–1.86 mA		
Geometry (cm^3^)	2.5 × 1.8 × 1.4	2.0 × 1.8 × 1.1	1.9 × 1.9 × 3	2.3 × 2.3 × 2	3 × 1.5 × 0.5
Weight	4.5 g	3.6 g	5.7 g	3.6 g	2.8 g
Power Cons.	—	—	35 mW	18.9 mW	
Experimental Arena	—	—	42 × 24 × 20 cm^3^		
Power Carrier	—	—	13.56 MHz		
Duration of Uninterrupted Experiment	3.5–4.2 hours	3–5.5 hours	5.7 hours	Unlimited	

## Methods

### 4-coil inductive link for wireless powering

EnerCage-HC2 is equipped with one driving coil at the bottom of the homecage and four resonators around it^24^. The Tx resonators are made of copper foils to achieve higher quality (*Q*) factor than the conventional wire-wound coils^24^. Since the nominal distance of 3 cm for implant in the rat abdominal cavity is typically lower than the 7 cm nominal distance of a headstage from the Tx coil, we optimized the Rx coils (*L*_3_ and *L*_4_) geometries in the implants accordingly to achieve highest power transfer efficiency (PTE) following the procedure in Mirbozorgi *et al*.^49^. In a 4-coil link, PTE can be found from^50^,

2 $${\eta }_{4-coil}=\frac{({k}_{12}^{2}{Q}_{1}{Q}_{2})({k}_{23}^{2}{Q}_{2}{Q}_{3})({k}_{34}^{2}{Q}_{3}{Q}_{4L})}{[(1+{k}_{12}^{2}{Q}_{1}{Q}_{2})(1+{k}_{34}^{2}{Q}_{3}{Q}_{4L})+{k}_{23}^{2}{Q}_{2}{Q}_{3}][1+{k}_{23}^{2}{Q}_{2}{Q}_{3}+{k}_{34}^{2}{Q}_{3}{Q}_{4L}]},$$

where *k*_*ij*_ is the coupling coefficient between coils *i* and *j*, *Q*_*i*_ is the quality factor of coil *i*, and *Q*_*4L*_ is the loaded quality factor of L_4_ in Fig. 2. The resulting Rx coils’ specs, shown in Fig. 6c, have been summarized in Table 2. The overall efficiency in the animal body from the source to load at the nominal distance of 3 cm from the bottom of the homecage was measured to be 19.2% and the power delivered to the load (PDL) was 18.9 mW.

**Table 2:** Specifications of the Tx and Rx coils used in 4-coil inductive link in a standard homecage.

Parameters	L_1_	L_21_, L_22_	L_23_, L_24_	L_3_	L_4_
Number of turns	3	1	1	4	6
Type of coil (Copper)	AWG 15	Foil tape	Foil tape	AWG 26	AWG 26
Inductance (µH)	5.46	0.88	0.94	0.87	0.69
Outer diameter(cm)	13	—	—	—	1.5
Inner diameter(cm)	12.8	—	—	—	0.8
Length(cm)	—	32	44	3	—
Width (cm)	—	22	22	1.5	—
Conductor width (cm)	—	25	25	—	—
Thickness (µm)	—	89	89	—	—
Diameter (mm)	1.45	—	—	0.4	0.4
Carrier Frequency	13.56 MHz				
Overall Efficiency (%)	19.2% @ 3 cm				

### Animal preparation and implantation surgery

Surgical procedures were performed under aseptic conditions at the UTRGV Animal Facility. Prior to implantation, each Lewis rat was placed into an induction chamber and subjected to gas anesthesia (5% Isoflurane with oxygen) until it became unconscious. Its maxillary central incisors were hooked into a gas mask through which it continued to receive small doses of anesthesia (2% Isoflurane). The rat body was secured on a surgery table and its body temperature was regulated with placement of a heat pad. The surgery locations, the right thigh and the right lower quadrant of the abdominal areas, were shaved and cleaned using a betadine scrub and isopropyl alcohol. An incision was made along the right thigh to expose the sciatic nerve and implant cuff electrodes. The incision continued to the right lower quadrant of the abdominal space to separate the abdominal skin from the abdominal wall for subcutaneous implantation of the WINeRS-8 implant.

The microwires of the cuff electrodes were penetrated vertically into the sciatic nerve and the polydimethylsiloxane (PDMS) tube guiding microwires was sutured around the nerve, proximal to the tibial and fibular nerves. The microwires coming out from the cuff electrodes were 1-inch long. The other end of the microwires were soldered on the WINeRS-8 implant. The 1-inch wire connection was long enough to split the devices in the two separate locations. The cuff electrodes were placed in the right hind leg and the transmitter was placed in the abdominal area. The WINeRS-8 implant, double-sealed by epoxy and PDMS with 0.5 mm thickness, was pocketed under the lower quadrant of abdominal area aligned to the midline of the body and sutured on the abdominal wall.

All procedures conformed to the Guide for the Care and Use of Laboratory Animals of the Institute of Laboratory Animal Resources, Commission on Life Sciences, National Research Council. They were reviewed and approved by the Institutional Animal Care and Use Committee at UTRGV and Georgia Tech.

### RF signal processing in SDR-based Rx

SDRs typically include amplifiers, filters, mixers, modulators/demodulators, ADC, control unit, and computer interface with many adjustable parameters, such as carrier frequency, bandwidth, gain, and modulation method, for flexible/programmable RF transceiver implementation. Because of this flexibility, SDRs have drawn both academic and industry interests for rapid implementation of radios needed for various applications^51^. The GNU radio is widely used as a development tool for research, education, and proof-of-concept prototyping due to its free open sources availability of many RF signal processing blocks^42^. In Fig. 5b, the parameters of RF center frequency, LNA gain, VGA gain, bandwidth, and sampling rate of ADC in two BladeRF SDRs^43^ are defined in the GNU radio. The ADC sampling rate was set to 36 MS/s, which is only 4 times higher than the uplink data rate of 9 Mbps, but still sufficient for demodulating the incoming RF signal. Digitized I and Q signals from individual SDRs are filtered by the first moving average filter of M = 2k to compare the averaged received RF power, *E*_*Avr1*_ and *E*_*Avr2*_, in real time. The digitized data stream from the SDR with stronger signal is selected as the received RF signal. A second moving average filter is used to alleviate the high frequency noise, before the signal is demodulated by adaptive threshold detection, using the output of the first moving average filter. The demodulated data is down-sampled by a factor of 4, and 13-bit preamble is found from the incoming data packet. Finally, the packet is sent to the C++ GUI to be unpacked and demultiplexed to construct individual recording channels, and to be displayed on the PC screen, all in real time (Supplementary Fig. S4).

## Electronic supplementary material

Supplementary figures

Supplementary movie 2

Supplementary movie 3

Supplementary movie 1
