# Supplementary material for: An Implantable Peripheral Nerve Recording and Stimulation System for Experiments on Freely Moving Animal Subjects
Source: Sci Rep. 2018 Apr 17;8:6115. doi: 10.1038/s41598-018-24465-1 (PMC5904113; doi:10.1038/s41598-018-24465-1)
Supplement: Supplementary file 1 — Supplementary figures [file 41598_2018_24465_MOESM1_ESM.docx]

**An Implantable Peripheral Nerve Recording and Stimulation System for Experiments on Freely Moving Animal Subjects**

# Byunghun Lee^1,3^, Mukhesh K. Koripalli^2^, Yaoyao Jia^1^, Joshua Acosta^2^, M. S. E. Sendi^1^, Yoonsu Choi^2^, and Maysam Ghovanloo^1*^

^1^Georgia Institute of Technology, School of Electrical and Computer Engineering, Atlanta, 30308, USA

^2^University of Texas, Rio Grande Valley, Department of Electrical Engineering, Edinburg, 78539, USA

^3^Incheon National University, Department of Electrical Engineering, Incheon, 22012, South Korea

^*^mgh@gatech.edu


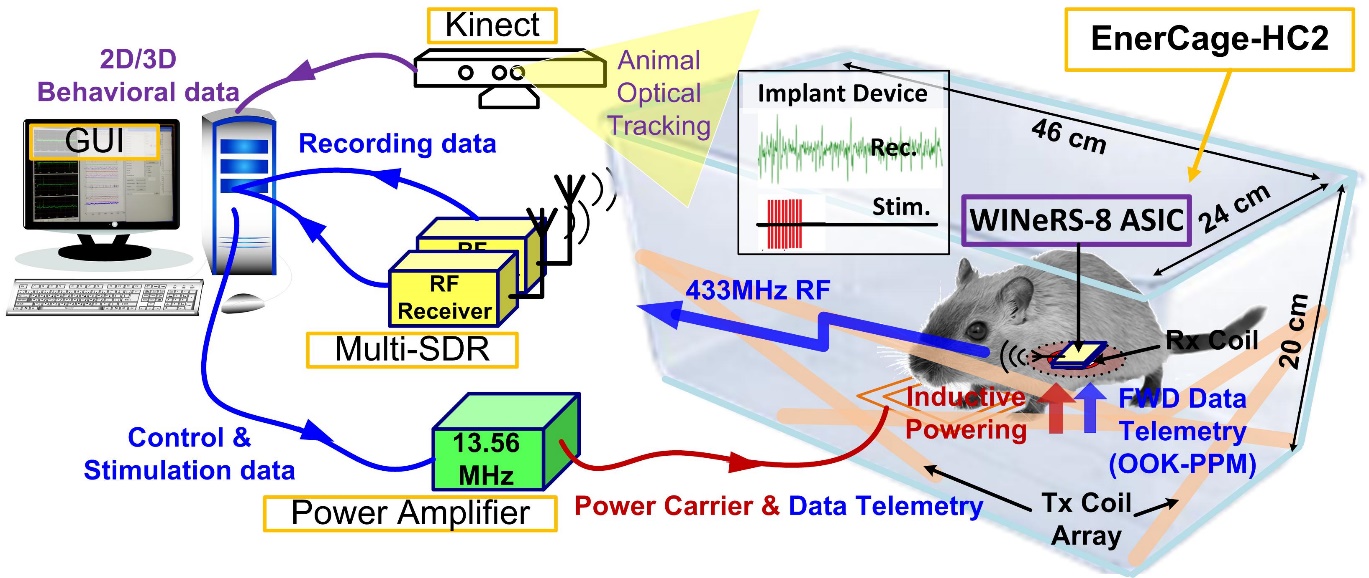


**Figure S1.** A conceptual view of power and data flows for inductively-powered wireless implantable neural recording and stimulation (WINeRS-8) system within the EnerCage-HC2 system, allowing for experiments involving long-term peripheral nerve recording and stimulation on small freely behaving animal subjects.


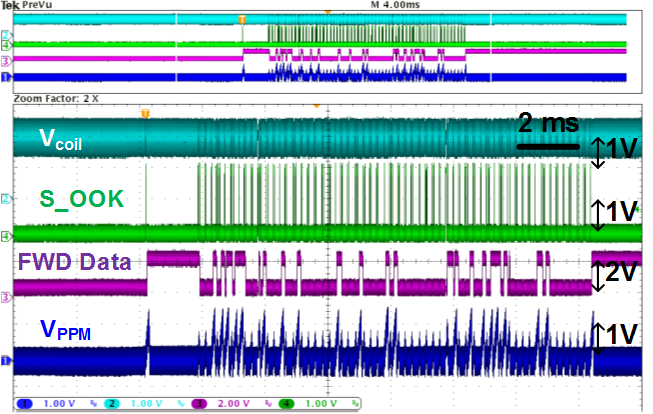

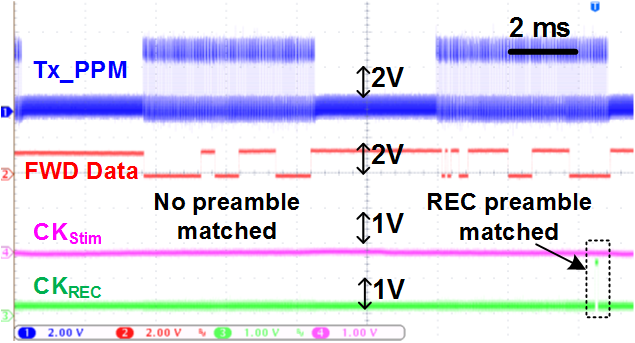


1. (b)

**Figure S2.** Measured waveforms of OOK-PPM near-field downlink data telemetry between WINeRS-8 and EnerCage-HC2 systems. (a) *V_coil_*, S_OOK, FWD Data, and *V_PPM_* in WINeRS-8 system (see Fig. 4a), and (b) Tx_PPM from EnerCage-HC2 system to trigger *CKREC* in WINeRS-8 system with matched preamble (see Fig. 4b).


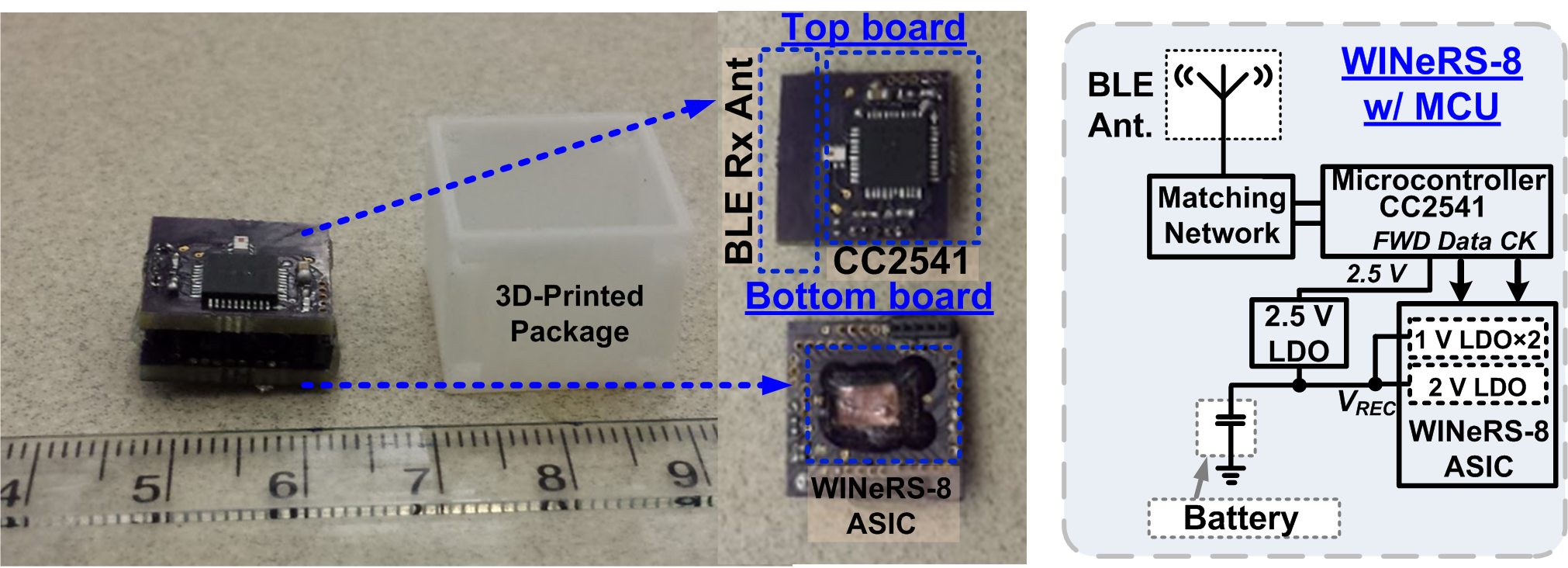


**Figure S3.** A battery-powered WINeRS-8 headstage equipped with a microcontroller (CC2541) for 2.4 GHz Bluetooth Low Energy (BLE), replacing OOK-PPM downlink data telemetry outside the EnerCage-HC2 system for an animal study to be conducted on a treadmill (Fig. 8b). An external 2.5 V LDO regulator is utilized to supply CC2541. A PCB antenna is implemented for the BLE communication at 2.4 GHz.


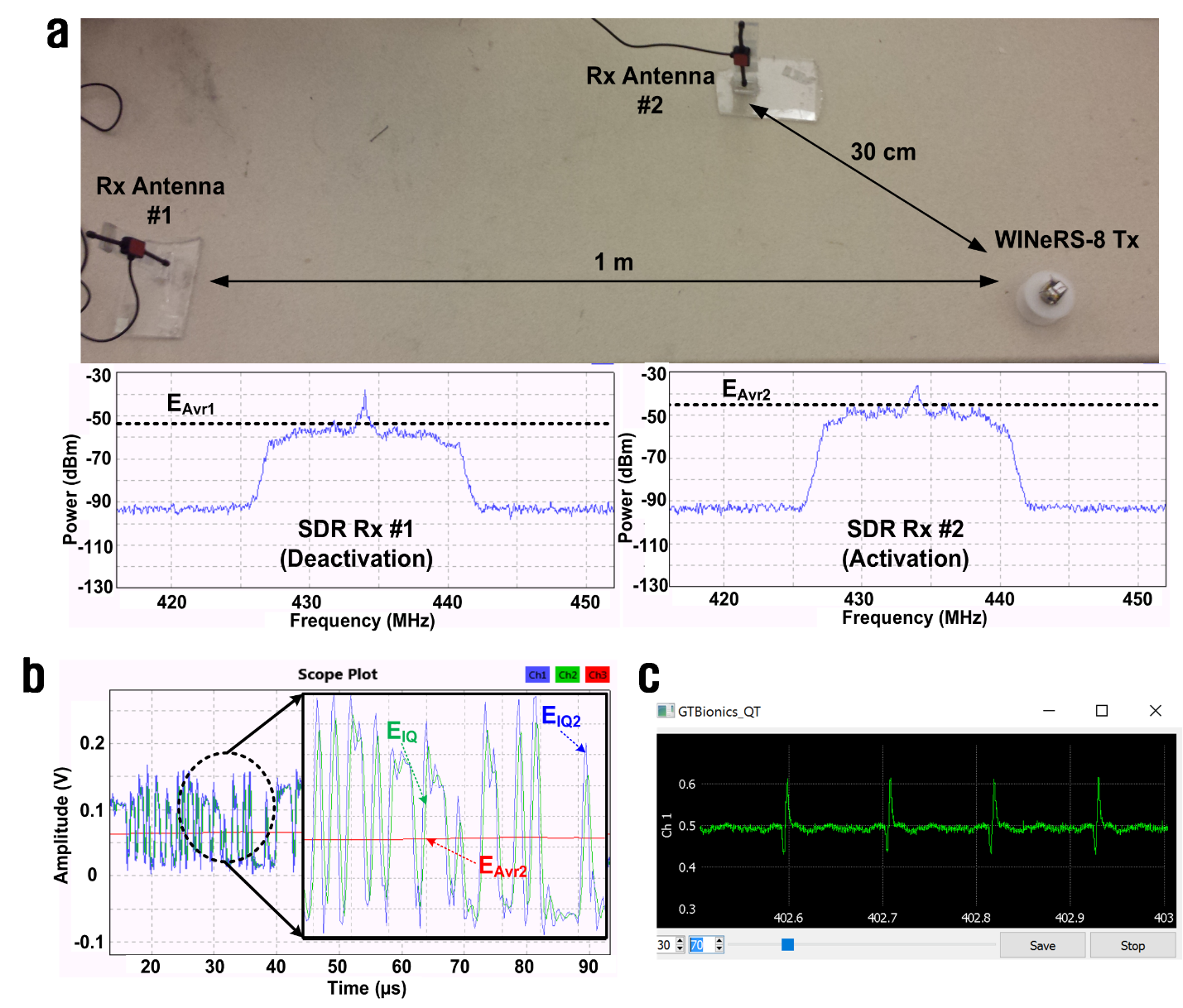


**Figure S4.** (a) Benchtop measurements on dual-SDR receivers with WINeRS-8 RF transmitter (Top) and received average power at two antennas, *E_Avr1_* and *E_Avr2_* (Bottom), (b) 36 MHz over-sampled transient RF signals after 1^st^ and 2^nd^ moving average filters in Fig. 5b, and (c) real time recovered and visualized signal of recording channel #1 in the customized GUI, developed in C++.
